# Current stress and poor oral health

**DOI:** 10.1186/s12903-016-0284-y

**Published:** 2016-09-02

**Authors:** A. Vasiliou, K. Shankardass, R. Nisenbaum, C. Quiñonez

**Affiliations:** 1 Faculty of Dentistry, University of Toronto, Toronto, ON Canada; 2 Department of Health Sciences, Wilfrid Laurier University, 75 University Ave West, Waterloo, ON N2L 3C5 Canada; 3 Centre for Urban Health Solutions, Li Ka Shing Knowledge Institute, St Michael’s Hospital, Toronto, ON Canada; 4 Dalla Lana School of Public Health, University of Toronto, Toronto, ON Canada

**Keywords:** Stress, Oral pain, General oral health, Dental insurance

## Abstract

**Background:**

Psychological stress appears to contribute to poor oral health systemically in combination with other chronic diseases. Few studies directly examine this relationship.

**Methods:**

Data from a cross-sectional study of 2,412 participants between the ages of 25–64 years old living in the City of Toronto between 2009 and 2012 were used to examine the relationship between current stress and two self-rated oral health outcomes (general oral health and oral pain). Dental care utilization and access to dental insurance were examined as effect modifiers.

**Results:**

A positive relationship between current stress and poor oral health was observed for both outcomes (oral pain coefficient 0.32, 95 % CI 0.26–0.38; general oral health coefficient 0.28, 95 % CI 0.19–0.36). Effects on oral pain were stronger for the uninsured, while effects on general oral health were stronger with decreasing socioeconomic position.

**Conclusions:**

Our findings suggest that individuals with greater perceived stress also report poorer oral health, and that this relationship is modified by dental insurance and socioeconomic position. These findings warrant a greater focus on the role of psychological stress in the development of oral disease, including how perceived stress contributes to health inequities in self-reported oral health status. Patients experiencing stressful lives may differentially require closer monitoring and more vigilant maintenance of their oral health, above and beyond that which is needed to achieve a state of health in the oral environment of less stressed individuals. There may be health promoting effects of addressing psychosocial concerns related to dental care - particularly for the poor and uninsured.

## Background

In the absence of policy changes to fully include dental health services in the Ontario Health Insurance Plan (OHIP), or through other social welfare programs, (such as Healthy Smiles Ontario) [1], more effective approaches to prevention are needed to maintain oral health status in some populations and to improve status in the general population. Whereas policy strategies to improve oral health in the population have traditionally targeted behavior change at the individual level by promoting brushing and flossing practices [2], more research is needed to understand the more distal and structural determinants of oral health.

The prevalence of poor dental health outcomes is usually markedly increased in populations of lower socio-economic position relative to those of higher socio-economic position, even after accounting for differences in access to care [3]. This has inspired growing interest in understanding the role of psychosocial factors in the development of diminished oral health status [4], including chronic stress (e.g., [5–8]).

### A systemic view of oral health and stress

In fact, poor oral health has long been theorized to cause the dysfunction of other critical physiologic systems [9]. A growing body of evidence has come to support the existence of such an “oral-systemic” relationship [10–12]. This relationship has been demonstrated for some diseases more so than others, including respiratory infections, osteoporosis, childhood obesity, cardiovascular disease, and type II diabetes [13–16]. A shared impetus for the development of both oral and systemic disease may be the presence of stress. As a common risk factor for both diseases of the oral cavity as well as for non-communicable diseases (e.g. cancer, cardiovascular disease, diabetes, and respiratory disease), the minimization of stress has become an integral component of novel systemic healthcare promotion techniques, such as the common risk factor approach [17].

Chronic stress is likely to contribute to the progressive, long-term development of oral disease through at least two distinguishable pathways. First, stress can motivate individuals to cope in unhealthy ways that foster oral disease (e.g., substance use, including illicit drugs, alcohol and tobacco, poor diet, and sedentary behavior). Second, chronic stress contributes to high allostatic load that can lead to the dysfunction of physiological systems critical to homeostasis, and thus, affect the underlying mechanisms of disease progression, more generally [18].

**Fig. 1:**
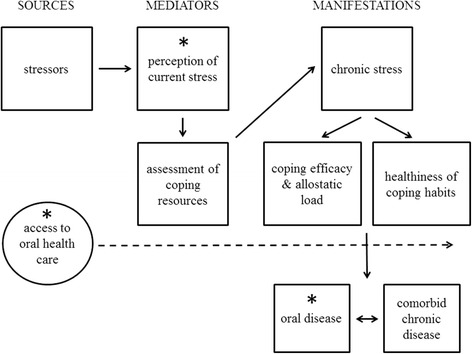
Conceptual framework of causal and moderating pathways linking the stress process to oral health practices and outcomes. Boxes indicate factors involved in the stress process (i.e., sources, mediators and manifestations). Straight lines indicate main causal relationships of interest. Circle with dashed line indicates a moderating effect of oral health practices

Our conceptual framework is described below and in Fig. 1, and many elements are elaborated on in Shankardass [18]. While stress is a concern of an array of disciplines, including psychology, sociology, psychoneuroimmunology, we adopt a transdisciplinary approach to defining chronic stress based on the stress process paradigm of Pearlin et al. [19]. They conceptualized stress as a process “in which demands strain on an individual’s ability to adapt - physiologically and emotionally - with implications for physiological and behavioural pathways” [19].

Individuals may experience potential *sources* of current stress, including people (such as children in the case of parents), places (such as densely populated intersections), and things (such as a paucity of money, food, and shelter). Typically, stressors can be categorized into one of four types: major life events (such as a death in the family), ambient strains (such as a concern for safety in the neighbourhood you live in), role strains (such as stress related to workplace hierarchy), and quotidian nature strains (stresses that result from activities of a repeated nature, such as a daily commute to work) [19].

Some factors may mediate whether or not the experience of stress currently translates into more chronic stress over time (*manifestations*), including whether or not they perceive certain stressors as threatening, and if so, manageable given resources at hand [19]. Then, where perceived current stress occurs, the coping behaviours used to deal with stressors mediate whether chronic stress manifests and harms oral health and general well-being in two main ways. First, if stressors associated with perceived current stress are not coped with in an effective manner, then chronic stress is more likely to occur. Allostatic load, a cumulative physiological impact of chronic stress, has been associated with periodontal disease [20], and some evidence indicates that this occurs due to increased inflammation [21]. However, the precise causal mechanisms remain somewhat unclear [22]. Second, regardless of how effective coping behaviours are, they may manifest as habits that are either healthy (e.g., exercise and relaxation, problem solving) or unhealthy (e.g., tobacco, alcohol and other drug use, poor oral maintenance) with respect to oral disease.

In this analysis we consider the general salience of our framework by investigating whether or not the perception of current stress is associated with self-reported poor oral health status among residents of Toronto, Ontario’s most populous city. We also examine whether this relationship is moderated by dental insurance and dental care utilization, since having greater access to oral health care (e.g., because of dental insurance or as reflected by actual visits to the dentist) may help to buffer the impact of chronic stress on poor oral health and other social and health outcomes.

## Methods

### Study design and instruments

We conducted a secondary analysis of the cross-sectional Neighbourhood Effects on Health and Well-Being (NEHW) study. The Research Board at St. Michael’s Hospital in Toronto, Canada provided ethics approval for this study. All participants provided written informed consent at the time of their interview and were compensated $50 for their participation. As described elsewhere in more detail, a three stage sampling strategy was employed to randomly sample households within a random sample of census tracts and neighbourhood planning areas in the City of Toronto [23].

Participants of the study were recruited between 2009 and 2012. Participants were eligible for the study if they were able to communicate in English, were between the ages of 25–64 years old, lived in their neighbourhood for at least 6 months, and were a resident of the home. Further details on study procedures can be obtained elsewhere [23].

The NEHW study collected data on neighbourhood and individual level factors that promote or diminish mental health using same-room face-to-face surveys in a total of 2,412 participants (response rate of 72 %; [24]) living in a random sample of 47 neighbourhoods throughout Toronto [23]. The survey included two self-rated oral health questions utilized as dependent variables in this analysis. General oral health was assessed using the question, “In general, would you say the health of your teeth and mouth is…?” (5-point Likert scale from 1 = “excellent” to 5 = “poor” health), while oral pain was assessed using the question, “In the past month, how often have you had any pain or discomfort in your teeth or gums? (4-point Likert scale from 1 = “often” to 4 = “never”). Therefore, higher scores indicate poorer oral health for both outcomes. Although not a direct measure of oral disease (see: [25]), self-reported oral health has been associated with greater chronic stress, depressive symptoms, and material hardship [26], so we expected it to be sensitive to perceived current stress.

Recency of dental care utilization was assessed using the question, “How long has it been since your last dentist visit?” (<1 year, 1- < 2 year, 2+ years), and access to dental insurance was assessed using the question, “Do you have insurance that covers all or part of your dental expenses?” (yes versus no).

Data were also available for several stress items, including some items from the Cumulative Adversity Interview (CAI) [27]. Higher levels of subjective stress based on the CAI have been associated with smaller grey matter volumes in parts of the brain [27]. Three items from the CAI were used in this study to capture a general index of whether or not participants perceived their lives as generally stressful at the time of the study (i.e., currently): “You’re trying to take on too many things at once”, “There is too much pressure on you to be like other people”, “Too much is expected of you by others”. We viewed the first item as indicating personal or work-related stress, and the other two as indicators of social stress. Each item was rated on a 3-point Likert scale (0 = “Not true”, 1 = “Somewhat true”, 2 = “Very true”) and an index was calculated by taking the average of the three items. Although this index has not been previously validated, we viewed it as measuring the level that participants perceived their lives as generally stressful at the time of the study (i.e., currently).

Other questions included in this analysis are age (years), sex (male or female), total household income (>$10,000, $10,000–$14,999, $15,000–$19,999, $20,000–$29,999, $30,000–$39,999, $40,000–$49,999, $50,000–$74,999, >$75,000), current employment status (indicator of any current employment), immigration status (Canadian born, immigrated ≤10 years ago, immigrated >10 years ago), high educational attainment (indicator of education beyond high school diploma). We also identified participants who were foreign born immigrants to Canada, i.e., not including those who were citizens through birth or parental descent. To parsimoniously test for the effect of these factors as confounders and moderators of the relationship between stress and oral health, an index of socioeconomic position was constructed by summing three indicator variables for high educational attainment, current employment, and high total household income (i.e., greater than or equal to $30,000); this resulted in an index with possible values ranging from 0 to 3.

### Data analysis

The crude and adjusted associations between perceived current stress and poor oral health outcomes were examined using univariate and multivariate linear regression models, respectively. All analyses were performed using IBM SPSS Statistics 22. The selection of confounders began with the use of theory to identify a list of candidates, including age, sex, socioeconomic position, foreign born, immigrant status, recency of dental care utilization and presence of dental insurance. Potential confounders were deemed appropriate for inclusion in the model building process if there was a statistically significant association with the oral health outcomes (alpha = 0.05). The final model was developed using a forward stepwise process using a cut-off for inclusion of a 10 % change in the coefficient for stress.

Moderation of the relationship between stress and oral health was examined by testing for statistical significance of multiplicative interaction terms in the final models. Given the exploratory nature of this analysis, we adopted a relatively broad alpha level for these tests of 0.10. Factors examined as potential moderators included age, sex, socioeconomic position, foreign born immigrant status, recency of dental care utilization and presence of dental insurance.

The assumption of a linear effect of stress on oral health status was tested by including dummy variables for quarter-point increase in the perceived current stress index (i.e., allowing a non-linear relationship to take shape). Plots of these models compared to a line of best fit (i.e., linear trend) did not indicate deviation from a linear trend (data not shown), and adjusted R-square values were also largely similar when the effect of stress was modeled as an exponential or cubic factor compared with linear models (data not shown).

Both rudimentary (via visual inspections of lines of best fit) and statistical (via R-square values) assessments of linearity were performed in an effort to detect whether the assumption of linearity was appropriate for the relationship between perceived current stress and oral health outcomes.

Sampling weights were derived previously [23] and utilized in all analyses to correct for any selection biases by comparing the distribution of sample characteristics using 2006 Census data for the City of Toronto.

## Results

Survey participants were 44 years of age on average (standard deviation of 11 years) and 48 % were male (Table 1). In general, participants were well educated (77.2 % had more than a high school education), wealthy (45.7 % had a total combined household income of greater than $75,000), and employed (71.6 % were currently employed). Almost 40 % of participants were born in Canada, while 15 % were recent immigrants and 46 % were long-term immigrants. Of all immigrant participants, 38 % were of a minority race/ethnicity. Almost 65 % of participants had some dental care insurance, while most participants (74 %) had visited the dentist within the previous year.

**Table 1:** Study participant characteristics

Characteristics	n (%)	Mean (SD)
Age (Years)		44.4 (10.7)
Male Sex	1163 (48.2)	
Reports of Pain or Discomfort in Teeth or Gums Over Past Month (1–4; 1 = Never, 4 = Often)		1.6 (0.86)
General Health of Teeth and Mouth (1–5; 1 = Excellent, 5 = Poor)		2.6 (1.1)
Perceived Current Stress Index (average of a - c)		0.67 (0.55)
a. You are trying to take on too many things at once (0–2; 0 = Not True, 2 = Very True)		0.89 (0.74)
b. There is too much pressure on you to be like other people (0–2; 0 = Not True, 2 = Very True)		0.41 (0.65)
c. Too much is expected of you by others (0–2; 0 = Not True, 2 = Very True)		0.69 (0.73)
Total Combined Household Income		
<$10,000	22 (1.0)	
$10,000–$14,999	87 (3.9)	
$15,000–$19,999	60 (2.7)	
$20,000–$29,999	231 (10.3)	
$30,000–$39,999	180 (8.0)	
$40,000–$49,999	188 (8.4)	
$50,000–$74,999	450 (20.1)	
>$75,000	1023 (45.7)	
Educational Attainment Greater Than High School Diploma? (Yes vs No)	1859 (77.2)	
Currently Employed? (Yes vs No)	2099 (87.2)	
Socioeconomic Position Index? (0–3)		2.4 (0.79)
Citizenship Status		
Canadian Born	947 (39.4)	
Non-Recent Immigrant	1106 (46.0)	
Recent Immigrant (≤10 Years in Canada)	351 (14.6)	
Has Dental Insurance That Covers All or Part of Dental Expenses? (Yes vs No)	1549 (64.3)	
Length Of Time Since Last Visit to the Dentist?		
<1 Year Ago	1782 (74.0)	
1–2 Years Ago	318 (13.2)	
>2 Years Ago	308 (12.8)	

The crude association between perceived stress and both perceived oral pain and perceived general oral health yielded similar positive and statistically significant (α = 0.05) beta coefficients (0.33 and 0.31, respectively) [Table 2, Model 1]. Age and sex were identified as confounders in the final model for both oral health outcomes, while household income was also a confounder of general oral health. While the effect of stress remained positive and statistically significant after adjusting for confounders, the coefficient was reduced by 10 % in the case of general oral health; whereas the effect on oral pain was relatively stable (3 % reduction) [Table 2, Model 2].

**Table 2:** Coefficients for perceived current stress index with oral health outcomes

	Model 1	Model 2
Oral Health Outcomes	Crude Current Stress Beta Coefficient (95 % CI)	Adjusted Current Stress and Oral Health Beta Coefficient (95 % CI)
Pain or discomfort in teeth or gums over past month	0.33 (0.27–0.39)	0.32 (0.26–0.38)
General health of teeth and mouth	0.31 (0.23–0.39)	0.28 (0.19–0.36)

Model 1 models the crude effect of the perceived current stress index. Model 2 is further adjusted age and sex for oral pain, and for age, sex and household income for general oral health

Table 3 examines whether the effect of the perceived current stress index was driven by any particular item. Coefficients for personal/work stress items were generally weaker than those for social stress.

**Table 3:** Comparison of coefficients for stress index components against oral health outcomes

	You are trying to take on too many things at once	There is too much pressure on you to be like other people	Too much is expected of you by others
Oral health outcomes	Coefficient (95 % CI)	Coefficient (95 % CI)	Coefficient (95 % CI)
Pain or discomfort in teeth or gums over past month	0.15 (0.10–0.19)	0.29 (0.22–0.32)	0.17 (0.13–0.22)
General health of teeth and mouth	0.13 (0.07–0.19)	0.17 (0.10–0.24)	0.19 (0.13–0.25)

**Fig. 2:**
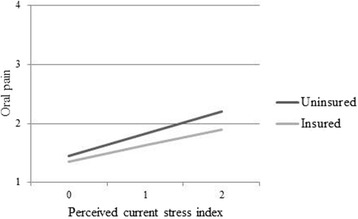
Predicted oral pain by perceived current stress for levels of dental insurance. Oral pain (1 – 4; 1 = Never, 4 = Often)

There was a statistically significant interaction between perceived current stress and dental insurance on oral pain (*p* = 0.09), where the effect of perceived current stress was slightly stronger for participants without dental insurance compared to the insured. Figure 2 describes the predicted levels of oral pain across levels of perceived current stress for insured and uninsured 44 year-old females who have a total household income of $40,000-$49,999. This model predicted a difference in oral pain of 0.31 where these insured and uninsured individuals are experiencing high perceived current stress.

**Fig. 3:**
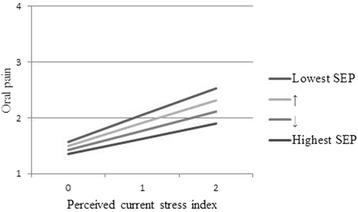
Predicted oral pain by perceived current stress for levels of socioeconomic position (SEP). Oral pain (1–4; 1 = Never, 4 = Often)

We also observed a statistically significant interaction between perceived current stress and socioeconomic position on oral pain (*p* = 0.06), where the effect of perceived current stress was stronger for participants of lower socioeconomic position. Figure 3 describes the predicted levels of oral pain across levels of perceived current oral stress for participants across four levels of socioeconomic position who are 44 year-old females with a total household income of $40,000–$49,999. This model predicted a difference in oral pain of 0.64 between individuals experiencing high perceived current stress with highest and lowest socioeconomic position.

## Discussion

We found that Toronto participants who reported greater perceived current stress in their lives also self-reported progressively poorer oral health and greater oral pain compared to those with lesser stress. The association with oral pain is novel, while our findings generally support other studies indicating a positive association between stress and poor oral health. Segura and Sheiham (1992) found a positive association between work-related demands and periodontal illness indicated by bleeding or pockets in a clinical exam [5]. Finlayson et al (2010) found positive associations between chronic stress and poorer self-rated oral health [26]; as did Armfield et al (2013), including a positive significant associated between chronic stress and untreated decayed teeth [8]. These findings indicate the salience of our framework linking perceived current stress to chronic disease, including oral disease (Fig. 1).

Some other studies have not observed associations between measures of stress and poor oral health (e.g., [7]). One explanation for inconsistent findings is that studies about stress are not properly accounting for the stress process and potential moderators of effects on oral health in the analysis of data. For example, associations observed in the study by Armfield et al (2013) were diminished in size and rendered non-significant in the case of untreated decayed teeth when models were adjusted for oral maintenance behaviours (i.e., tooth brushing) [8]. We argue that such factors may be on the causal pathway (e.g., [6]) as part of the response to perceived stress, and therefore should not be included in statistical models as potential confounders of effects of stress on oral health. Dental care utilization was also included as a confounder in that analysis and contributed to the diminished effect of stress on poor oral health [8]; whereas our findings suggest that this factor may actually moderate this association.

We were not able to clarify whether current stress (i.e., at the time of the study) reflected generally more stressful lives, (i.e., chronic stress); nor could we measure much of the inherent behavioural and physiological process that ultimately contribute to oral disease pathology (as indicated by Fig. 1). Future studies should test hypotheses that involve more components of our framework, including the efficacy and healthfulness of coping behaviours, the experience of chronic stress and biomarkers of allostatic load, and comorbid chronic disease outcomes. While subjective measures of stress are likely to be good indicators of manifestations on health and well-being [27], future studies should examine effects on a wider range of specific oral disease end-points to help further clarify the precise mechanism that links stress to oral health.

The harmful effect of perceived current stress on oral pain was more pronounced for participants without dental insurance and of lower socioeconomic position. It is not unexpected that dental insurance conditioned the relationship between perceived current stress and oral pain since dental services are not publicly insured for residents of Toronto under Canada’s Medicare program. This pattern could indicate that those without insurance are not receiving adequate dental care (i.e., have unmet needs); for example, because they cannot afford to pay for service out-of-pocket [28–31].

The moderating effect of socioeconomic position was stronger than that observed for dental insurance. This relative difference could be explained if individuals of lower socioeconomic position experience a multitude of barriers to health. For example, this population may experience: 1) poorer access to adequate dental care given unemployment or employment in a position with miserly health benefits [32, 33]; 2) greater chronic stress and coping in ways that are more damaging to their oral health (e.g., tobacco use, alcohol use, consumption of junk food); and 3) greater risk of other chronic diseases, which may impact oral disease via the oral-systemic relationship.

In addition to water fluoridation, current preventative measures for oral health rely largely on the agency of individuals to maintain oral health practices and on education and early screening in clinical and other settings. For example, the Toronto Public Health Unit implements free dental screening in both public and non-public schools for children from Junior Kindergarten to Grade 8, as well as provides educational programs such as the Community Oral Health Outreach Program in an effort to teach individuals about dental health and disease prevention strategies [33]. Our findings may motivate future studies to investigate the impact of stress, dental insurance, and socioeconomic position on dental health outcomes - if a strong link is established, these current interventions are unlikely to be sufficient to prevent oral disease. To the extent that the relationships between stress, oral pain, dental insurance and socioeconomic position are causal, strategies to improve prevention include greater promotion of psychosocial health, broadened accessibility of dental care and greater promotion of social (read: income) equality.

The apparent importance of psychosocial causes of oral disease suggests that the Canadian status quo of excluding dental care services from Medicare simply because the causes of oral disease are easy to prevent (e.g., by large-scale treatment interventions such as fluoridation, and individuals practicing oral health promotion, such as brushing and flossing) [32] is not defensible. Thus, efforts to improve access to dental care as a way of buffering the impact of stress on oral health may be needed; particularly for the uninsured and those of lower socioeconomic position. In lieu of the Federal Government requiring dental care services to be insured under Medicare, other incentives for dentists in Toronto, Ontario to provide affordable care for the uninsured are needed. In addition, social welfare programs could be introduced to broaden access to dental services. For example, the Provincial Government recently introduced the Healthy Smiles Ontario program to provide dental insurance to children of lower income families, and this program could be expanded to cover all lower income Ontarians.

Our study participants were drawn from a random sample of neighbourhoods in Toronto, which strengthens the generalizability of our findings to adults living in the city. While psychological stress can be challenging to measure accurately, our findings are based on an index based on diverse types of stress, which strengthens the internal validity of the findings. Our study was limited by its reliance on self-reported measures of perceived oral health and current stress, making the results subject to recall and social desirability biases. Our study was also limited by cross-sectional data collection; although it could be argued that poorer oral health status is not likely to lead participants to report higher levels of the indicators used to measure stress in this study. Still, in order to better understand the mechanisms linking psychological stress to oral disease, longitudinal study designs should be employed in future studies.

## Conclusions

Our findings suggest that individuals with greater perceived stress also report poorer oral health, and that this relationship is modified by dental insurance and socioeconomic position. These findings may warrant greater attention be paid to the role of psychological stress in the development of oral disease, including as a cause of social inequalities in oral health, and health inequity, more generally. More research is needed to explain the relationship between perceived current stress and oral health and to inform the design of interventions for the uninsured and those disadvantaged in other ways.

## Abbreviations

NEHW: Neighbourhood effects on health and well-being
SEP: Socioeconomic position
